# An update on clinical trials for chemoprevention of human skin cancer

**DOI:** 10.20517/2394-4722.2022.99

**Published:** 2023-02-27

**Authors:** Victoria Jiminez, Nabiha Yusuf

**Affiliations:** 1 Heersink School of Medicine, University of Alabama at Birmingham, Birmingham, AL 35294 USA.; 2 Department of Dermatology, Heersink School of Medicine, University of Alabama at Birmingham, Birmingham, AL 35294 USA.

**Keywords:** Skin cancer, chemoprevention, melanoma, non-melanoma skin cancer, chemopreventive agents

## Abstract

The pathophysiology of skin cancer is complex, with multiple factors contributing to its development. The proactive treatment of skin cancer has been investigated in the form of chemoprevention of cutaneous malignancies in clinical trials. Chemoprevention is the use of natural or pharmacologic agents that prevent or reverse skin cancer development. Multiple trials have arisen over the past decades to explore the efficacy of specific agents to halt the progression of UV radiation damage. This comprehensive review article aims to assess clinical trials performed with chemopreventive agents for melanoma and nonmelanoma skin cancers. The following compounds were most often used in these trials: nicotinamide, retinoids, polyphenolic antioxidants, COX-2 selective inhibitors, non-steroidal anti-inflammatory drugs, difluoromethylornithine, and 5-fluorouracil. Many agents show promise in their ability to prevent nonmelanoma skin cancer formation, with few melanoma trials demonstrating efficacy. The chemoprevention efforts aimed at skin cancer are complex; current and future trials will be instrumental in identifying therapeutic agents that pose efficacy in halting cancer development and assessing whether long-term administration is tolerable.

## INTRODUCTION

Skin cancer is the most prominent malignancy worldwide, affecting every ethnicity, socioeconomic background, demographic cohort, geographic region, and age group^[1]^. The American Academy of Dermatology estimates that one in five Americans will develop skin cancer in their lifetime^[1,2]^. These cancers are subdivided into two broad categories: malignant melanomas and nonmelanoma skin cancers (NMSC). Malignant melanomas are the most frequent cause of skin cancer-related death, and it is estimated that about 7650 people are expected to die of melanoma in the U.S. in 2022^[3]^. The NMSC category is further delineated into two major subtypes: basal cell carcinomas (BCC) and squamous cell carcinomas (SCC) and it is estimated that 2–3 million cases occur each year in the world^[4]^. Environmental exposures such as ultraviolet radiation (UVR) are highly correlated with skin cancer development^[4]^. Educational initiatives to prevent exposure to risk factors for skin cancer and timely detection of these lesions are essential for preventing cutaneous malignancies and their progression.

The pathophysiology of skin cancer is complex and multiple factors contribute to the development of precancerous and cancerous lesions. With both melanoma and NMSC, the most common underlying etiology of malignancy formation is UVR-induced damage from sunlight exposure^[1]^. These rays mediate DNA damage by mutating tumor suppressor genes necessary for the maintenance of cellular homeostasis and a regulated life cycle. They also cause immunosuppression, inflammatory responses, and oxidative stress, leading to the dysregulation of skin cells and the development of malignancies^[1]^. These various cell damage mechanisms possess many routes that lead to homeostatic disruption in skin cells, making this type of cancer challenging to prevent and treat.

UVR can be subdivided into ultraviolet A (UVA) rays and ultraviolet B (UVB) rays, which exist along different wavelengths. These types of waves most frequently reach the earth’s surface in the form of UVA, with only 1%-10% of them existing as UVB^[4]^. UVA plays a role in carcinogenesis of the skin’s stem cells, while UVB radiation induces keratinocyte DNA damage, upregulation of inflammatory pathways, and tumor formation^[5,6]^. Dysregulated DNA repair processes lead to inhibition of apoptosis, expansion of mutated keratinocytes, and initiation of skin malignancies^[4,5]^. Chronic exposure to UVR is the most influential risk factor for developing actinic keratoses, cancer precursors. Additionally, more than one severe sunburn in childhood from sunlight and UVR exposure results in a 2-fold increase in melanoma risk^[7,8]^.

Immunosuppression induced via UVR is a contributing factor to carcinogenesis, frequently resulting in the development of skin cancers. Tumor formation occurs by diminishing antigen-presenting cell function, inducing immunosuppressive cytokine production, and altering delayed-type hypersensitivity reactions^[4,9]^. Due to impaired homeostatic functions in skin immunity, tumor formation has been associated with pro-inflammatory reactions via the arachidonic acid pathway. Upregulation of prostanoid production and cyclooxygenase 2 (COX-2) expression has been shown to increase mouse and human skin carcinogenesis, where these levels are low and maintained in normal skin^[10]^. Immunosuppression leading to skin cancer formation via other mechanisms has also been observed, such as in transplant patients who receive immunosuppressive therapies, allowing immune susceptibility to cutaneous malignancy formation^[11]^. Ultimately, multiple factors contribute to the development of skin cancer via mechanisms of free radical formation, DNA damage, alteration of homeostatic genes, and dysregulation of immune function, leaving the skin susceptible to the growth of malignancies.

Skin cancers often are not identified until their progression to solid tumors that are visible to the naked eye. While most are caught in early stages prior to metastasis, some aggressive forms of skin cancer such as melanoma may have already invaded the lymphatics or other parts of the body when a patient presents with a visible lesion. Thus, there has been a growing interest in recent years in the development of prophylactic agents to prevent skin cancer formation in patients believed to be at high risk for developing these malignancies. Many clinical trials have aimed to identify whether these chemopreventive agents are efficacious in halting malignancy formation and progression. In this review, we seek to provide an updated summary of chemopreventive agents that have emerged in recent decades through various trials and studies, as well as highlight future horizons in skin cancer treatment. This review was conducted via literature search using PubMed and included relevant articles using the search terms “chemoprevention”, “squamous cell carcinoma”, “basal cell carcinoma”, “melanoma”, and “skin cancer”. Pertinent trials and studies related to chemopreventive mechanisms and potential efficacious agents were included for discussion in this review.

## BACKGROUND ON CHEMOPREVENTION OF SKIN CANCER

Skin cancer most typically manifests in the later stages of life. The median ages for the presentation of melanoma, BCC, and SCC are 65, 69, and 75 years of age, respectively^[3,12]^. With UVR exposure over a lifetime, prevention efforts are limited in that people of all ages can be at risk for sun damage accumulated early in life that may not present in the form of visible cutaneous malignancies until decades later. There has been a demonstrated need for developing interventions that are used in the time period following damaging UVR exposure and before the development of skin cancers^[12]^.

**Figure 1.**
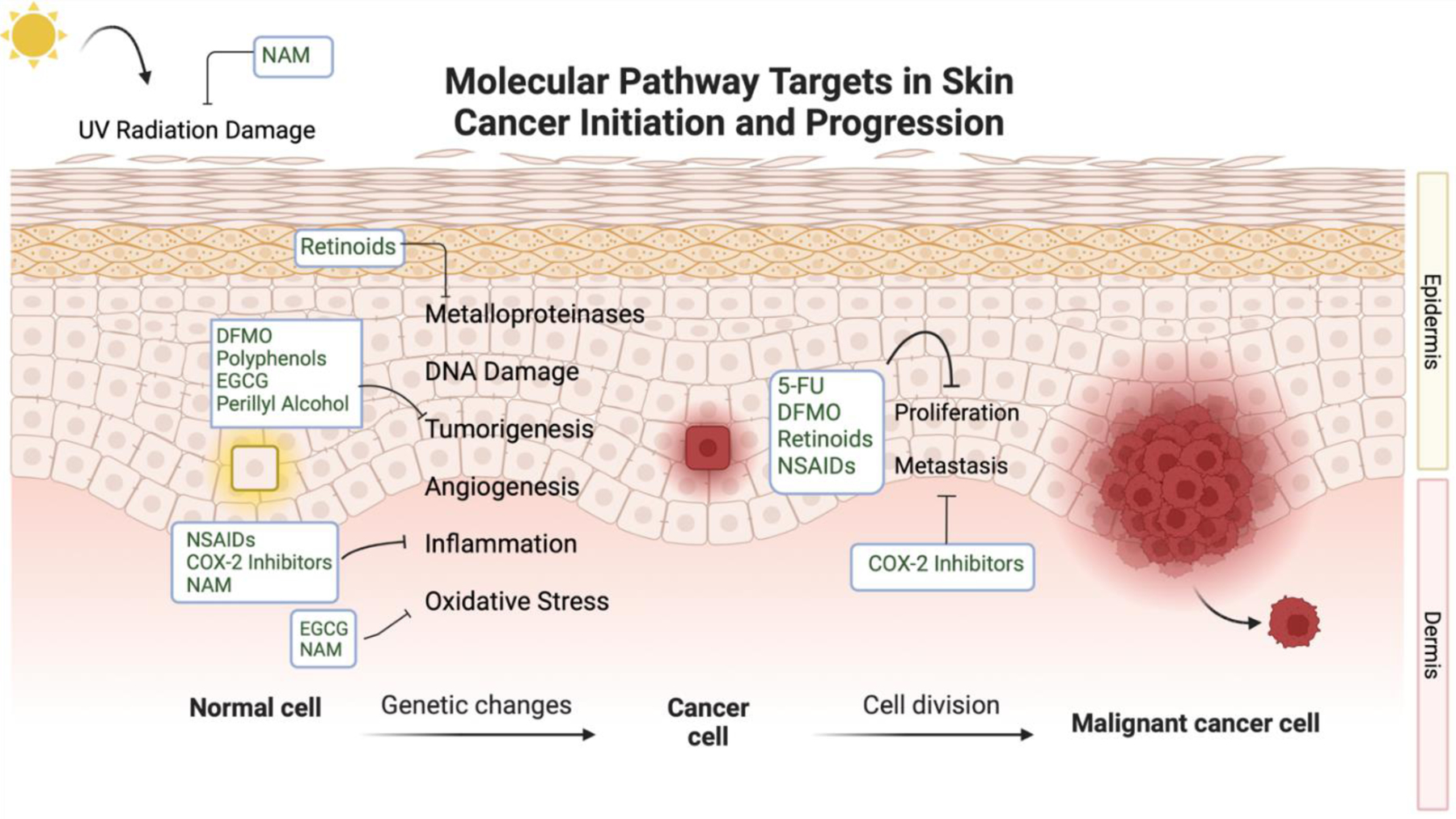
Molecular Pathway Targets in Skin Cancer Initiation and Progression Created with BioRender.com

Chemoprevention has been posed as a solution and is defined as using natural or pharmacologic agents that prevent or reverse skin cancer genesis^[12]^. Chemopreventive agents that pose promising effects often target pathways brought upon by UV radiation-induced tumorigenesis. Molecular targets in these transduction mechanisms include inflammatory cytokines, cyclooxygenase-2, prostanoids, melanocortin one receptors, oxidative stress contributors, and many others^[10,13,14]^. Benefits to these specific targeting mechanisms include low toxicity and directed action towards malignant proliferating cells. Many agents involved in regulating UVR signal transduction to halt skin cancer progression have been tested in clinical trials. The major compounds assessed in these trials include nicotinamide (NAM), retinoids, difluoromethylornithine (DFMO), polyphenolic antioxidants from green tea extracts, 5-fluorouracil (5-FU), COX-2 inhibitors such as celecoxib, and non-steroidal anti-inflammatory drugs (NSAIDs)^[10,12,15]^. Trials have aimed to assess the efficacy of oral and topical formulations in their potential to halt tumor formation pathways while maintaining low rates of adverse effects. Other agents that also pose benefits for chemoprevention have been investigated in cellular and animal studies, but have not yet been developed in the context of human trials. A summary of the chemopreventive agents and the pathways these compounds target during skin cancer development can be found in Figure 1.

These treatments are plausible for populations considered medium to high risk for developing skin malignancies. This includes patients with a history of high amounts of sun exposure, pre-malignant skin markers such as actinic keratoses, numerous or invasive previous skin cancers, immunosuppression history, and organ transplant recipients^[16]^. Criteria for risks of cutaneous malignancy formation include lesion location, size, borders, de novo *vs*. recurrent disease, underlying immunosuppression, prior radiation, and pathologic subtypes^[17]^.

Skin cancer accounts for a significant cause of morbidity in people who have considerable immunosuppression. Populations especially at risk for the development of skin cancer include immunosuppressed organ transplant recipients (OTRs). This group’s risk for developing NMSCs is increased by approximately 10–250 fold^[18]^. Immune function impairment in OTRs following chronic use of immunosuppressive medications causes an inability to eradicate cells with precancerous changes in addition to direct carcinogenic effects of these agents on cancer-initiated cells^[18]^. A few successful chemopreventive agents that have been used in OTR patients who are at high risk for the development of skin cancer include retinoids, cyclooxygenase inhibitors, and ornithine decarboxylase (ODC) inhibitors such as DFMO^[18]^.

Treatment intervention for skin cancer lesions varies in the form of surgical removal, radiation therapies, and systemic therapies such as immune checkpoint inhibitors^[17]^. While surgical removal is the mainstay of initial treatment for skin cancers without distant metastatic disease, patients become more vulnerable to surgical interventions with age. Skin cancer is most prominent in the elderly population, and with aging comes increased rates of comorbidities, functional losses, cognitive impairment and decreased wound healing ability^[19]^. A recent study in 2022 found that hypofractionated radiation therapy is a safe and efficient treatment for elderly patients with SCC^[19]^. It has been shown that SCC has overexpression of the epidermal growth factor receptor pathway, suggesting this pathway is a possible treatment target in the future^[17]^. Regarding basal cell carcinomas, dysregulation of the sonic hedgehog pathway is seen in these tumor types and emerging inhibitors such as Vismodegib and Sonidegib show promise^[17]^. Recent advancements have tremendously increased recurrence-free and overall survival in patients with melanoma through the relatively new monoclonal antibody immune checkpoint inhibitors and targeted therapies (BRAF/MEK inhibitors)^[17]^. For example, ipilimumab (CTLA-4 inhibitor) and pembrolizumab (PD-1 inhibitor) have significantly reduced the risk of recurrences in trials and increased overall survival benefits in their use as adjuvant therapies in patients who completed surgical resection of melanoma lesions^[17]^. However, systemic therapies also pose a risk for toxicities which elderly patients are more susceptible to. While recent advances in treatment modalities have instrumentally improved skin cancer outcomes, there still lies a need for prophylactic agents to prevent their development to avoid invasive interventions. It has been estimated that approximately 60% of people with a history of developing one NMSC will be diagnosed with a second within ten years^[20]^. With high skin cancer recurrence rates, new chemoprevention modalities pave a new horizon in treating patients at high risk for malignancy development and recurrence.

## CLINICAL TRIALS OF SKIN CANCER CHEMOPREVENTION

**Figure 2.**
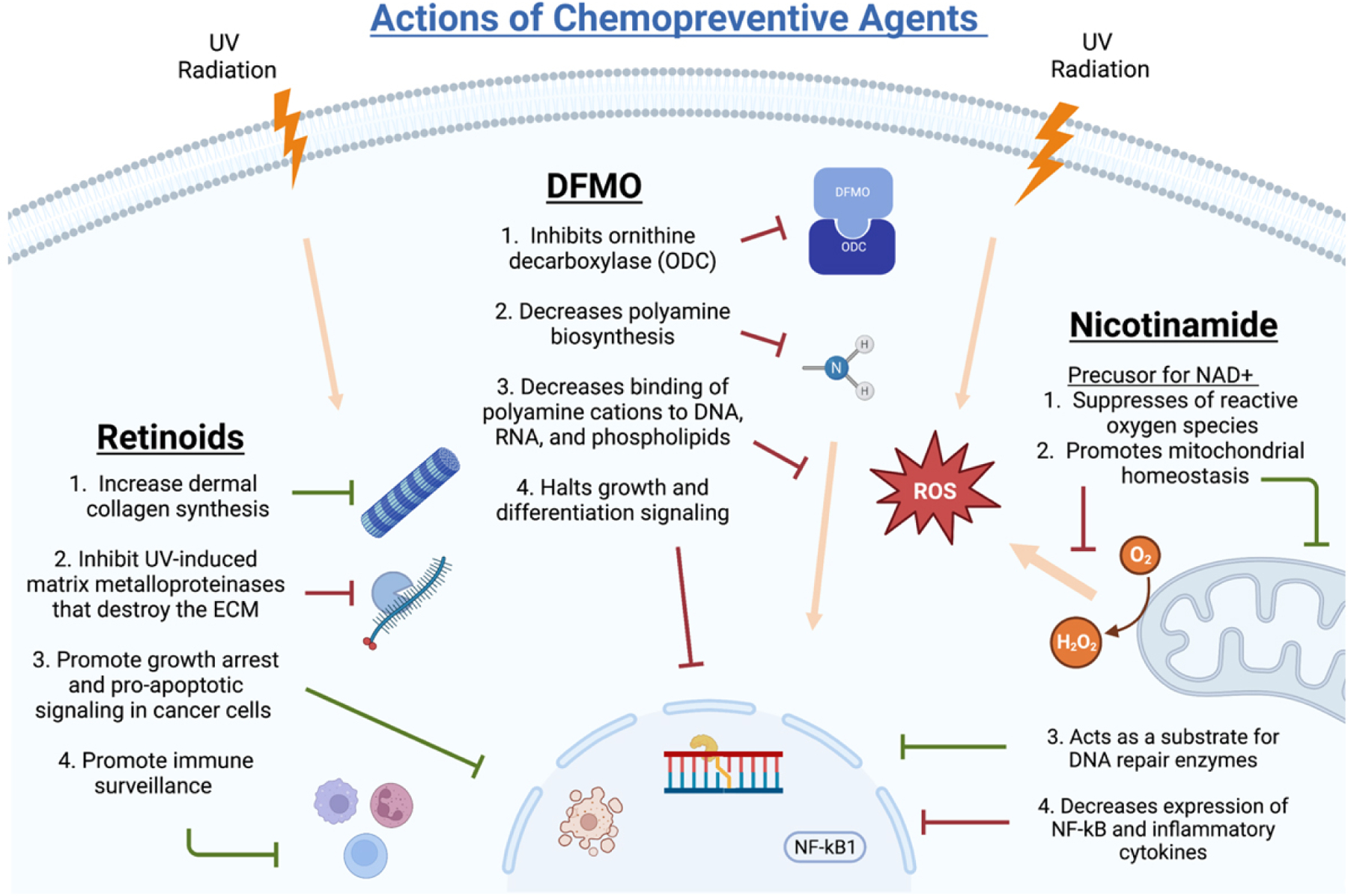
Detailed Mechanisms of Retinoids, DFMO, and Nicotinamide Chemoprevention

Chemopreventive agents that have shown promise in vitro and UV mouse models have been explored in humans through clinical trials. Potential new agents are selected from leads of basic science and epidemiology. Compounds with approved preclinical toxicology profiles advance to phase I and phase IIa clinical trials. After this, larger phase IIb and multicenter phase III trials are performed before final approval of efficacious agents in human populations^[15]^. Studies examining the chemopreventive activity of agents tested in trials have shown varying results, warranting current and future trials to establish which agents are safe and efficacious. Carefully selected target populations and identifying biomarkers with prognostic and predictive value are necessary to evaluate these therapeutics^[21]^. Limiting factors of clinical trial investigation include the rarity of cancer endpoints in populations, the large population sizes required for data reliability, length of time for carcinogenic progression, sun exposure, and subject compliance to long-term treatments with frequent and high dosing regimens^[15]^. Feasibility of the dietary and pharmacologic agents used in chemoprevention is difficult and accounts for significant challenges in past and future trials. The controversies surrounding the subject of chemoprevention are largely based on these clinical trial limitations and speculation of proven efficacy. Trials and studies that have most recently been discussed in the literature include the following agents: nicotinamide, COX-2 inhibitors/NSAIDs, retinoids, DFMO, and 5-FU^[16]^. The following sections describe these agents in detail and highlight clinical trials and studies related to their chemopreventive abilities. A summary of the main chemopreventive trials mentioned in this review can be found in Table 1 and detailed mechanisms of NAM, DFMO, and retinoids can be found in Figure 2.

### Nicotinamide trials

Nicotinamide (NAM) is a water-soluble form of Vitamin B3 (niacin) that also serves as a precursor for many cellular metabolism pathways in the form of nicotinamide adenine dinucleotide (NAD+)^[22]^. High NAD+ suppresses reactive oxygen species production and promotes mitochondrial homeostasis and cell survival^[22]^. Centrally involved in DNA repair mechanisms, NAM is a substrate for the DNA repair enzyme poly(ADP)ribose polymerase that acts to repair UV-mediated nucleotide damage^[23]^. NAM also possesses anti-inflammatory roles by decreasing the expression of NF-kB, a well-known pro-inflammatory transcription factor^[22]^.

Studies have demonstrated nicotinamide’s therapeutic action for inflammatory skin conditions such as bullous pemphigoid and acne vulgaris^[24]^. Evaluation of its use in animal models also lends promise to its ability to combat immunosuppression by UV radiation. In one of the first studies to investigate NAM in chemoprevention, Gensler *et al*. treated UV-irradiated mice with nicotinamide twice weekly and saw prevention of immunosuppression and skin tumor induction by reducing tumor incidence from 75% to 42.5%^[25]^. Overall, NAM increased NAD+ levels circulating in the blood, reduced immunosuppression in cutaneous areas of UV damage, and reduced inflammation in both cancerous and precancerous lesions^[22,26]^.

Over the past few decades, there has been a rise in evidence that NAM administration in humans might play a role in preventing cancer pathogenesis. NAM has been investigated in multiple phase III clinical trials of skin cancer prevention, which lend efficacy towards its use as a chemopreventive agent. It also has beneficial potential in its safety against toxicity, and cost-effectiveness and has been well tolerated in trials.

In 2012, a phase II clinical trial brought attention to nicotinamide’s ability to treat pre-cancerous lesions. Oral administration of NAM significantly reduced the incidence of actinic keratoses (AK) in high-risk individuals compared to placebo^[27]^. Furthermore, NAM has notably expressed clinical value in a 2015 phase III randomized trial of NAM by Chen *et al*., commonly referred to in the literature as the ONTRAC trial^[28]^. This trial was conducted in a cohort of 386 subjects labeled as high-risk patients due to the inclusion criterion of having at least 2 NMSCs in the previous five years^[28]^. Those given 500 mg of nicotinamide twice daily for twelve months had the rate of new NMSCs decline by 23% compared to the placebo group^[28]^. Specifically, the new BCC rate decreased by 20%, the new SCC rate decreased by 30%, and actinic keratoses decreased by 11%^[28]^. The results of this trial support the notion that oral NAM is safe and effective in reducing the recurrence of AKs, BCCs, and SCCs^[28]^. Neurocognitive function was also assessed in this trial but yielded no significant findings^[29]^. Upon cessation of NAM usage after a six-month follow-up period, there was no significant decrease in recurrence rates, suggesting that NAM administration is effective only on a daily regimen, without long-term effects after discontinuation. Thus, the argument arises of whether all chemopreventive agents must be used continuously for optimal efficacy. For each individual therapeutic, oral and topical agents warrant thorough investigation to assess for long-term adverse effects from their continual administration.

The usefulness of NAM has also been investigated recently in trials specific to immunosuppressed populations. Chen *et al*. also conducted a phase II-controlled chemoprevention trial in renal transplant patients in 2016^[30]^. With the same treatment of 500 mg oral NAM, there was a 16% reduction in AKs and a 35% reduction in NMSCs^[30]^. However, results were not significant due to a small sample size of 22^[30]^. The limitations of clinical trial abundance and reliability are especially prominent in this population of patients who have received solid organ transplants due to difficulty in the recruitment of large cohorts. A recent case-control study administered oral nicotinamide to a group of 38 patients who received either a kidney or liver transplant, and NAM was found to be effective in reducing rates of NMSCs and AKs compared to controls^[31]^. Of the 18 treated patients with a significant decrease in AK size, 42% showed complete clinical regression^[31]^. This further supports NAM’s value in chemoprevention for halting the development of NMSCs.

While nicotinamide reduces incidences of nonmelanoma skin cancers and actinic keratoses, no trials have been conducted on its use for melanoma chemoprevention. It has been hypothesized that the photoprotective effects of NAM against melanoma induction pathways, such as DNA damage and UV-mediated immunosuppression, suggest potential in its usefulness for treatment, similar to results gathered from NMSC trials^[23]^. In the ONTRAC study mentioned previously, six *in situ* and four invasive melanomas were reported during the NAM intervention period between both treated and control groups^[28]^. There were no significant differences between the thickness and invasiveness of lesions between the groups, suggesting limitations to the chemopreventive effects of NAM in melanoma reduction^[23,28]^. However, individuals were recruited based on the incidence of NMSCs, and patients with a history of melanoma in the previous five years were excluded^[23,28]^. Thus, to accurately assess NAM’s role in melanoma prevention, a group of high-risk for melanoma would need to be treated in trials^[23]^. Overall, the ONTRAC trial and other clinical studies suggest chemoprevention with nicotinamide clinical trials is warranted for high-risk melanoma individuals. A summary of NAM trials can be found in Table 1.

### COX-2 inhibitor trials

Cyclooxygenase (COX) inhibitors, commonly used as anti-inflammatory agents, have demonstrated promise in their potential as chemopreventive agents. The enzyme cyclooxygenase is central to prostaglandin production and inflammatory pathways in the body. Nonsteroidal anti-inflammatory drugs (NSAIDs) inhibit this enzyme and are known for their analgesic, antipyretic, and platelet-inhibiting properties^[12]^. Some NSAIDs inhibit COX-1 and COX-2 enzymes, leading to toxicity from loss of COX-1 action. Thus selective COX-2 inhibitors pose as better candidates for chemopreventive agents^[32]^. Studies have reported that an increase in COX-2 enzyme and, therefore, prostaglandin E_2_ levels are correlated strongly with cancer development and metastases^[12,33]^. Regarding the skin, expression of COX-2 has been observed to be stimulated by UVB radiation, increasing the incidence of cutaneous inflammatory pathways^[12,34,35]^.

Studies in mice models have suggested a relationship between COX-2 inhibition and decreasing cancer pathogenesis. Compared to controls, one leading COX-2 inhibitor, celecoxib, decreases multiplicity and increases tumor latency in UVR-exposed mice. It is effective in both low and high doses, converted to human dosages as 200 mg and 400 mg, respectively^[12,36]^. Other mouse model studies have proven celecoxib to reduce UV-induced tumorigenesis, prostaglandin synthesis in the epidermis, tumor yield, and skin tumor formation in mice^[12,13,37]^. COX-2 is upregulated in tumors and suggests that the arachidonic acid/COX-2/eicosanoid pathway has a significant role in photocarcinogenesis^[32,37]^. Treatment of squamous cancer cells in vitro with COX-2 inhibitors inhibits cell growth^[32,38]^.

When applied to humans, the effectiveness of oral celecoxib as a chemopreventive agent has been investigated in both oral and topical formulations. A double-blind, placebo-controlled, randomized clinical trial evaluated 240 subjects with a previous history of 10–40 AKs. After receiving 200 mg of oral celecoxib or placebo twice daily for nine months, no significant differences were found in the number of new AKs between the two study arms^[39]^. While fewer AK lesions were present in the celecoxib group, the lack of significant data suggested that celecoxib may effectively prevent SCCs and BCCs. Applying topical NSAIDs to areas of the skin at risk for the development of AKs and NMSCs is a point of interest. COX inhibitor diclofenac is an established treatment by the Food and Drug Administration (FDA) for actinic keratoses, lesions prominently associated with squamous cell carcinoma development. Randomized placebo-controlled trials have affirmed its efficacy and demonstrated that topical diclofenac gel could induce a 60%-80% complete response in AK lesion clearance^[40]^.

Regarding melanoma, COX-2 is expressed in malignant melanomas and is correlated with its development and progression^[40,41]^. In vitro studies have found at least a 3-fold increase in COX-2 levels in different melanoma cell lines^[42]^. Additionally, a recent study analyzed COX-2 levels in lymph nodes with melanoma metastases and found that high COX-2 expression (> 10%) reduced progression-free survival by almost three years^[42]^. One trial for patients with metastatic melanoma treated subjects with autologous dendritic cells, human telomerase reverse transcriptase, and tumor lysate to generate an immune response on a vaccine basis^[42]^. Meanwhile, these patients were also treated with cyclophosphamide, IL-2, and celecoxib. The overall survival rate of patients increased compared to a previous trial without cyclophosphamide and celecoxib; however, results cannot be solely attributed to the action of celecoxib^[43]^. Additionally, a case series of 27 patients with incurable metastatic melanoma who received celecoxib treatment obtained spontaneous regression^[42]^.

A recent phase II clinical trial evaluated the antiproliferative potential of oral aspirin associated with programmed cell death protein 1 (PD-1) or pembrolizumab and cytotoxic T-lymphocyte-associated protein 4 (CTLA4) or ipilimumab inhibitors in patients with unresectable stage III or stage IV melanoma^[42]^. The primary outcome of this study found that 52.2% of participants at 12 weeks confirmed complete response (CR) or partial response (PR) of melanoma per the Response Evaluation Criteria in Solid Tumors (RECIST)^[44]^.

Aspirin is a nonselective NSAID that suppresses prostaglandin E_2_ (PGE_2_) and activates adenosine monophosphate-activated protein kinase (AMPK) to inhibit colony formation, cell motility, and pigmentation while also reducing growth in some melanoma tumors *in vivo*^[45]^. Clinical trials are warranted, which evaluate the ability of COX-2 inhibitors and NSAIDs as the sole therapeutic in order to determine their chemopreventive value in human subjects with both NMSCs and melanoma.

The use of COX-2 inhibitors in immunosuppressed OTR populations poses potential risks regarding their negative effect on kidney graft and cardiovascular functions. Still, topical preparations of COX-2 inhibitors may be beneficial with no risk. Further research is warranted on cyclooxygenase chemoprevention in these populations. A summary of COX-2 trials can be found in Table 1.

### Retinol/Retinoid trials

Retinol, also known as Vitamin A, is a fat-soluble vitamin essential to many biological processes. Its use in skin aging is prominent as its topical application promotes dermal collagen synthesis, prevents the degradation of collagen, and inhibits UV-induced matrix metalloproteinases, which are enzymes that mediate extracellular matrix degradation^[46]^. Concerning cancer development, retinoids act at the nuclear level to cause growth arrest and apoptosis in rapidly dividing tumor cells. They also promote immune surveillance and are involved in the maturation of keratinocytes, and topical administration generates thickening of the skin. One derivative of retinol, tretinoin, has been used topically for the treatment of AK. Studies have found significant decreases in AK lesions and sizes of lesions upon treatment with retinol^[47,48]^.

It has been suggested that isotretinoin, a retinoid derivative commonly used to treat cystic acne, is the most effective retinoid for preventing NMSC in high-risk patients in clinical trials^[47]^.

A positive chemoprevention trial on NMSCs involving retinol administration was conducted with 2297 subjects with moderate to severe AKs. Administration of oral retinol (25,000 IU) significantly reduced squamous cell carcinomas but not basal cell carcinomas^[32,49]^. Another study of 719 subjects with BCC or SCC were administered retinol, isotretinoin, or placebo, and no differences in new NMSC development or tumor multiplicity were found^[50]^. A multicenter clinical trial on subjects with two or more BCCs was administered low doses of isotretinoin and also yielded no significant results^[51]^. In this trial, not only was isotretinoin ineffective in reducing the number of BCCs, but treatment was also correlated with significant systemic adverse effects^[52]^. It has been suggested that these studies indicate that retinoids are more effective in the early stages of skin carcinogenesis, rather than in high-risk patients with multiple previous NMSCs or more progressed diseases^[32]^. However, when patients with the inherited disease xeroderma pigmentosum were administered isotretinoin, a rapid and significant reduction in the incidence of new skin cancers was observed^[47]^.

Acitretin, another vitamin A derivative, has been proposed for chemoprevention. A prospective, randomized, double-blind trial tested skin cancer prevention effects of a 2-year treatment with acitretin on 70 patients with a history of more than 2 NMSCs within five years of trial onset^[51]^. Subjects were randomized to a placebo or acitretin 25 mg orally five days/week with outcomes of the rate of new NMSC development. The resulting data did not have a statistically significant reduction in the rate of new primary NMSCs of treated subjects^[52]^. However, umbrella testing indicated a significant trend favoring acitretin for the incidence of new NMSC, time to new NMSC, and total NMSC counts. These results were suggested to have been the result of low statistical power^[52]^.

In special populations of solid organ transplant recipients, retinoids have been identified to be protective in the development of NMSC^[18]^. Systemic retinoid use has reduced the appearance of AKs and SCC development risk in the immunocompetent population. Similar studies in transplant subjects have yielded reductions in AKs, but there is no clear consensus on whether it prevents SCC development. Multiple trials have generated contrasting results on whether different retinoid therapeutics significantly affect incidences of SCC in transplant populations^[53–56]^. However, the use of retinoids as chemopreventive agents after transplantation is recommended in those at high risk for NMSC development, as there have been multiple trials and studies supporting the notion that retinoids such as acitretin and etretinate show favorable results for chemoprevention in high-risk organ transplant recipients (OTRs)^[57]^. An open randomized crossover trial evaluating the efficacy of acitretin for NMSC chemoprevention in renal allograft recipients was performed on a cohort of 23 patients. After the first year of drug-free evaluation, subjects were given 25 mg of acitretin daily for one year and the number of SCCs in patients while on acitretin was significantly reduced compared to the drug-free period^[56]^. A similar trend was exhibited in BCCs; however, it was not significant. Of note is the toleration of this medication for this trial in that 9 of the participants withdrew from the trial due to adverse effects from this medication^[56]^. Further evaluation is needed to assess the plausibility of long-term, frequent dosing regimens with these derivatives and whether alternating days of dosage or drug holidays could yield higher tolerability.

Drawbacks to retinoid use include teratogenicity and the necessity for long-term treatment. These agents are highly teratogenic because women must wait up to two years after discontinuing certain oral retinoid medications before considering pregnancy^[53]^. Adverse effects reported from long-term administration of certain derivatives include increased mucositis and skin toxicities^[51]^. Additionally, a rebound effect has been observed in the frequency of skin cancers upon discontinuation of systemic retinoids, thus necessitating long-term treatment with strict adherence^[53]^. A summary of retinoid trials can be found in Table 1.

### Difluoromethylornithine trials

DFMO is an irreversible inhibitor of ODC, an enzyme essential in polyamine biosynthesis and the production of amino acids. ODC activity increases in UV-medicated skin carcinogenesis observed in vitro^[58]^. Polyamines are crucial to cell growth, proliferation, and tumor promotion when dysregulated^[59]^. Epithelial carcinogenesis of skin, breast, and colon tissue has been linked to levels of polyamines^[60]^. Further, studies show that many tumor promoters increase ODC activity and a number of preneoplastic conditions and tumor samples show high levels of ODC, suggesting that ODC may act as an oncogene^[59]^. Under this rationale, DFMO has been reported as a possible chemopreventive agent due to its inhibition of this enzyme.

DFMO effectively reduces actinic keratoses and basal cell carcinomas in immunocompetent populations^[61]^. Multiple studies have proven ODC to be a molecular target for the chemoprevention of both SCCs and BCCs in experimental animals^[62,63]^. Adverse effects of these medications include their associations with dose-dependent hearing loss, although a clinical trial found it to be well tolerated with evidence of mild ototoxicity^[64,65]^.

Multiple clinical trials have been performed, supporting the validity of DFMO as one of the leading agents for chemoprevention. A randomized, placebo-controlled phase IIb trial found DFMO to reduce skin polyamine concentrations by 21%, as well as the total number of AKs on the forearms of patients with at least ten distinct AKs^[15]^. In Another phase II trial, topical DFMO reduced AK number, suppressed polyamines, and reduced p53 proteins^[66]^. The combination of topical diclofenac, an NSAID, and topical DFMO has demonstrated efficacy in chemoprevention ability for SCCs^[67]^.

In 2010, subjects with a history of skin cancer were enrolled in a phase III clinical trial with daily administration of oral DFMO^[65]^. 291 participants with a history of NMSCs were randomized to placebo or oral DFMO for 4–5 years. The primary endpoint, new NMSCs, was not significantly different between subjects taking DFMO and placebo. While there were no significant differences in squamous cell carcinomas between groups, there was a substantial decrease in new basal cell carcinomas among the treated arm^[65]^. These results may be because the Hedgehog signaling pathway is essential to basal cell carcinogenesis. DFMO also reduces mRNA expression of sonic hedgehog and glioma-associated transcription factors^[62,65]^. Conclusions of this trial lend interest to the plausibility that sensitivities to ODC inhibition vary between squamous and basal cell carcinogenesis.

One clinical trial enrolled 159 subjects and randomized them to 90 days of topical DFMO, topical diclofenac (an NSAID), or both medications^[67]^. Polyamine levels did not vary significantly between the groups or from levels before the study. COX-2 expression did decrease over time, but did not vary significantly between the groups. The results of this study hypothesized that diclofenac and DFMO might also affect COX-2 levels with cross-talk between the polyamine and COX-2 pathways^[66]^. This trial was limited in its lack of a placebo arm. The combination of DFMO and NSAIDs are effective in cancer prevention at other sites, such as colonic adenomas, and further trials are warranted to address the efficacy of this combination^[67,68]^.

In OTR populations, Carbone *et al*. conducted a phase I trial of 18 subjects to investigate DFMO on skin polyamine levels and TPA-induced ODC toxicity^[69]^. Significant inhibition of ODC activity was achieved, and polyamine levels were suppressed, suggesting that DFMO might be an effective chemopreventive agent in skin cancer for transplant recipients. However, trials in these populations are needed to further explore its safety and efficacy^[69]^. A summary of DFMO trials can be found in Table 1.

### Phytochemicals: epigallocatechin-3-gallate (EGCG) trials

The transcription factor activated protein-1 (AP-1) is another important player in pathways of skin cancer development, and its activation leads to UV-induced tumor production. Compounds that target this transduction pathway have been tested as chemopreventive agents, such as green tea polyphenols. These formulations have antioxidant properties, and epidemiologic studies have illustrated associations between green tea consumption and decreased risk of cancer development^[70]^. Epigallocatechin-3-gallate (EGCG) is an active antioxidant, serving as the most abundant polyphenol. With its anti-inflammatory potential, EGCG has also been correlated with suppression of the COX and lipoxygenase pathway, commonly targeted by other chemopreventive agents^[71]^.

Inhibition of human tumor cell line growth has been demonstrated by EGCG, even in melanoma. *In vivo* mice, studies of topical application with this compound found significant reductions in the multiplicity and volume of UV-induced skin tumors with no visible toxicity or loss of UV-induced immunosuppression^[72]^. Regarding the AP-1 pathway, investigations have observed that EGCG significantly inhibits AP-1 transcriptional activation in HCL14 keratinocytes^[15]^. Similar results in human keratinocyte cell lines showed EGCG inhibition of UVB-induced activation of AP-1^[73]^. It has also been hypothesized that EGCG downregulates inflammasome and nuclear factor (NF)-kB activity and even abolishes tumor necrosis factor receptor-associated factor 6 (TRAF6) activity in melanoma by preventing migration and invasion of cells^[74,75]^.

Few clinical trials have assessed the therapeutic chemopreventive abilities of EGCG. One study in human subjects found that topical green tea polyphenols confirmed their protective activities against UV-induced erythema^[76]^. However, a recent single-blind clinical trial of 50 healthy adults who had supplementation of oral green tea extract with vitamin C did not have a significant reduction in skin erythema or leukocyte infiltration^[77]^. An area of disagreement arises when assessing the efficacy of oral versus topical EGCG. Outcomes in mice have shown that tumor-reducing outcomes were obtained by topical EGCG, but oral formulations were ineffective^[71]^. Hypotheses for this result are due to inadequate EGCG supplies in the skin following oral ingestion^[71]^. Clinical trials have been tested using EGCG as a chemopreventive agent. In a double-blind phase II clinical trial, 51 volunteers with AK were given topical EGCG for a 12-week period. No significant differences among treatment and placebo groups were found for the prevention of NMSCs, possibly due to poor bioavailability and lack of activity in the formulation^[78]^.

Many other phytochemicals have been studied in vitro and pose potential benefits for application to human skin cancer models. These agents are biologically active compounds derived from herbal products and plants, are highly tolerated, and are cost-effective^[78]^. In addition to EGCG, they include (6)-gingerol, caffeic acid phenethyl ester, capsaicin, curcumin, eugenol, caffeic acid, genistein, luteolin, silymarin/silibinin, resveratrol, ursolic acid, allyl sulfides, and indole-3-carbinol. Their targets include various pathways leading to tumor formation, including oncogenes, oxidative stress, and UV radiation damage^[78]^. Another naturally occurring agent in soil called selenium has antioxidant properties and has shown chemoprotective effects when used in mice with UV-induced melanoma^[79]^. Similar to polyphenol efficacy in skin cancer prevention via the UVB/AP-1 signal transduction pathway, a monoterpene derived from fruits and vegetables called perillyl alcohol blocks it as well. This compound inhibits UVB-induced AP-1 transcriptional activation and tumorigenesis when applied topically to mice. Other agents exerting effects on the AP-1 signaling pathway include salicylates blocking the MAPK cascade, and several synthetic retinoids^[15]^.

Recognized limitations to phytochemicals in skin cancer include formulation and delivery for optimal bioavailability, adverse effects, and lack of evidence for the recommendation for their use in preventing cutaneous malignancies^[78]^. While no clinical trials have been completed on these remaining compounds in humans with skin cancer, they provide a promising outlook for the future of chemoprevention. A summary of phytochemical trials can be found in Table 1.

### 5 fluorouracil trials

5-FU is an antimetabolite drug widely used for cancer treatments, particularly colorectal cancer. Thymidylate synthase is an active enzyme in forming thymidine nucleotides in cancer cell DNA. 5-FU exerts its effects via thymidylate synthase inhibition and incorporation of its metabolites into RNA and DNA. 5-FU has been suggested to treat actinic keratosis and types of basal cell carcinomas. However, for chemoprevention purposes, a large clinical trial demonstrated the ability of 5-FU to prevent the development of squamous cell carcinomas.

The Veterans Affairs Keratinocyte Carcinoma chemoprevention Trial is a high-impact trial that has frequently been referred to in the literature^[79]^. This trial was a randomized, double-blind, placebo-controlled trial of topical fluorouracil for chemoprevention of keratinocyte carcinomas (KC), also known as NMSC, in 932 subjects with a history of at least 2 KCs in the past five years^[80]^. Application of fluorouracil (5%) or vehicle control cream on the face and ears twice daily for 2–4 weeks was assigned upon randomization. No difference was found between treatment groups in time to a first keratinocyte, basal cell, or squamous cell carcinoma. However, during the first year of the study, 5 participants (1%) in the fluorouracil group developed squamous cell carcinoma *vs*. 20 (4%) in the control group, yielding a 75% risk reduction^[79]^. There were no significant effects on basal cell carcinoma risk reduction or keratinocyte carcinoma risks. Conclusions of this study lend rationale that fluorouracil substantially reduces surgery for squamous cell carcinomas for one year on the face and ears, and a reduction in KC was observed^[80]^.

Synergistic effects of calcipotriol, a topical thymic stromal lymphopoietin inducer, and 5-FU were assessed in a randomized, double-blind trial with 131 subjects^[81]^. Topical administration consisting of 0.005% calcipotriol ointment and 5% 5-FU cream was found to suppress skin cancer development and reduce the number of AKs by 87.7% in the treatment group versus 26.3% in the control group. This trial concluded that this combination optimally activated CD4+ T cell-mediated immunity against actinic keratoses and, potentially, skin cancer^[81]^.

In immunosuppressed populations, capecitabine, a pro-drug for 5-FU, was tested at low doses in solid transplant recipients and found incidences of SCCs and AKs to be significantly reduced with manageable toxicity^[82]^. A summary of 5-FU trials can be found in Table 1.

Recent advances in clinical trials and investigations lend interest to the efficacy of chemopreventive interventions as future pharmaceuticals for the prevention of skin cancer in high-risk populations. With the increased understanding of the pathophysiology of cutaneous malignancy development, molecular markers continue to be identified as targets in these pathways. Limitations to this review include the possibility that our literature search did not encompass all relevant trials and studies related to chemoprevention in skin cancer in recent years. Clinical trial identification numbers were not available for all trials mentioned in the review as some were international studies, older trials, or we could not locate the trial number. Additionally, data from ongoing or recently completed clinical trials are not yet available and may yield new findings that are not reported in this review. The lack of concrete evidence for chemopreventive abilities of many proposed agents hinders the ability to draw strong conclusions about their efficacy.

## CONCLUSION

The chemoprevention efforts aimed at skin cancer are complex; future trials will be instrumental in identifying therapeutic agents that pose efficacy in halting cancer development while also being tolerable for long-term administration. There have been limited trials assessing chemoprevention in both melanoma and skin cancer in solid organ transplant immunosuppressed patients, and more are needed in this cohort to prevent skin cancer mortality in these populations.

## Figures and Tables

**Table 1:** Major human clinical trials for chemoprevention of both melanoma and nonmelanoma skin cancers

Trial Agent	Description	Target for Outcomes	Dosage	Results

Nicotinamide	Phase II randomized, double-blind, controlled	AK	500 mg oral QD* Or 500 mg oral BID**	Oral NAM effectively reduces number of AK in high-risk individuals^26^
Nicotinamide	Phase III randomized double blind, controlled	NMSC	500 mg oral BID**	Oral NAM is safe and effective in reducing recurrence of AKs, BCCs, and SCCs^27^
Nicotinamide	Phase II, randomized, double blind, controlled	NMSC in renal or liver transplant recipients	500 mg oral BID**	Oral NAM overall reduced AKs and NMSCs, no significance due to small sample size^29^
Celecoxib	Randomized, double blind, placebo-controlled	AK	200 mg oral BID**	Overall reduction in AK lesions, but lacks significant data^38^
Diclofenac	Randomized, placebo-controlled trials	AK	Topical diclofenac gel 1% BID**	Diclofenac is well tolerated and can induce a 60–80% complete response in AK lesion clearance^39^
Celecoxib, autologous dendritic cell vaccination, IL-2, cyclophosphamide	Phase II trial	Metastatic Melanoma	200 mg oral QD*	Number of patients obtaining stable disease more than doubled 6-month survival compared to previous trial without cyclophosphamide and celecoxib^42^
Aspirin, Pembrolizumab, Ipilimumab	Phase II, open label study	Stage III/IV Melanoma	IV Pembrolizumab, IV Ipilimumab, oral aspirin BID**	52.2% of participants experienced complete response or partial response^43^
Retinol	Randomized, double-blind, controlled	NMSC	25,000 IU oral retinol QD*	Daily supplementation of retinol is effective in preventing SCC, but not BCC^48^
Isotretinoin	Multicenter trial	NMSC	10 mg isotretinoin QD*	Isotretinoin is ineffective in treating BCCs and long-term administration caused toxicity^50^
Retinol or Isotretinoin	Randomized, double blind, placebo-controlled	NMSC	25,000 IU oral retinol or 5–10 mg oral isotretinoin	No beneficial effects were found regarding NMSC prevention by retinol or isotretinoin^49^
Acitretin	Prospective, randomized, double-blind, placebo-controlled	NMSC	25 mg oral acitretin QD*	No signficiant reduction in NMSC with acitretin use, however, umbrella testing indicated significant trend favoring acitretin usage^51^
Acitretin	Open, randomized, crossover trial	NMSC in renal allograft recipients	25 mg oral acitretin QD*	Significant reduction in the number of SCCs with similar trend in BCC reduction, acitretin associated with systemic toxicity^55^
DFMO	Phase IIb randomized, placebo-controlled	AK	Topical DFMO	Significant reduction in skin polyamines, reduced p53 protein, and total number of AK^15,65^
DFMO	Phase IIb, randomized	Change in average nuclear abnormalities in sun-damaged skin	Topical DFMO BID**, Topical diclofenac QD**, or topical DFMO + topical diclofenac	Addition of topical DFMO to topical diclofenac did not enhance treatment against cutaneous sun damage^66^
DFMO	Phase III, randomized, double-blind, placebo-controlled	NMSC	500 mg/m(2) oral DFMO QD*	No significance in new NMSC reduction between treatment groups, but significant difference in new BCC^64^
DFMO	Phase I	ODC activity in solid organ transplant recipients	0.5 g and 1.0 g DFMO QD*	DFMO is safe and well tolerated in OTR patients and suggests possible efficacy in NMSC prevention^68^
EGCG	Phase II, randomized, double-blind, placebo-controlled	AK	Topical EGCG	No significance in prevention of NMSCs^82^
5-fluorouracil	Randomized, double-blind, placebo-controlled	NMSC/KC	Topical 5% fluorouracil to face and ears BID**	5-FU reduces surgery for SCC and reduces NMSC/KC treated with Mohs surgery^79^
5-fluorouracil + calcipotriol	Randomized, double-blind,	AK	Topical 5% fluorouracil + topical 0.005% calcipotriol BID**	Synergistic effects of calcipotriol and 5-FU optimally activate CD4+ T cell mediated immunity against AKs and potentially skin cancer^80^

* QD (once daily)

** BID (twice daily)

AK: actinic keratosis; NAM: nicotinamide; NMSC: nonmelanoma skin cancer; BCC: basal cell carcinoma; SCC: squamous cell carcinoma; DFMO: difluoromethylornithine; EGCG: epigallocatechin-3-gallate; 5-FU: 5-fluorouracil; KC: keratinocyte carcinoma.
